# Combination of Active Components Enhances the Efficacy of *Prunella* in Prevention and Treatment of Lung Cancer

**DOI:** 10.3390/molecules15117893

**Published:** 2010-11-04

**Authors:** Liang Feng, Xiao-Bin Jia, Jun Jiang, Mao-Mao Zhu, Yan Chen, Xiao-Bin Tan, Feng Shi

**Affiliations:** 1 Key Laboratory of Delivery Systems of Chinese Materia Medica, Jiangsu Provincial Academy of Chinese Medicine, Jiangsu, Nanjing, 210028, China; E-Mails: wenmoxiushi@163.com (L.F.); 2 Biotechnology Laboratory of Chinese Medicine, Macau University of Science and Technology, Macau, China; E-Mail: xuyan9323@126.com (J.J.); ychen202@yahoo.com.cn (Y.C.); jakytam2005@hotmail.com (X.-B.T.); shifeng_1985_wcl@163.com (F.S.); 3 Analysis Center, Rudong County Grain Bureau, Jiangsu, Nantong, 226400, China; E-Mail: zhumaomao823@126.com (M.-M.Z.); 4 State Key Laboratory of Food Additive and Condiment Testing, Zhen Jiang Entry-Exit Inspection and Quarantine Bureau, Jiangsu, Zhenjiang, 212008, China

**Keywords:** efficacy material basis, *Prunella*, active components combination mode, prevention, lung cancer

## Abstract

The efficacy of *Prunella* extracts in the prevention and treatment of lung cancer has been attributed to different components. In this study, an "active components combination model" hypothesis was proposed to explain the anti-tumor activity of *Prunella*. The efficacy of *Prunella* extracts from different regions was compared *in vitro* and *in vivo*, and the TNF-α activity in serum of tumor-bearing mice was also evaluated. High performance liquid chromatography (HPLC) was used to analyze the extracts and identify 26 common peaks. *Prunella* samples from different regions were classified by the cluster analysis method; both *P. vulgaris* L. from Bozhou and *P. asiatica* Nakai from Nanjing, which had the highest activities, were further divided into different classes. Six peaks from the HPLC analysis were very similar, and were identified as caffeic acid, rosmarinic acid, rutin, quercetin, oleanolic acid and ursolic acid. The total ratio of these compounds in *Prunella* from Bozhou and Nanjing were 1.0:14.7:3.9:1.0:4.4:1.4 and 1.0:14.8:4.0:0.8:5.6:1.8, respectively. Total triterpenes and total phenols in *Prunella* were separated by macroporous resin purification for activity studies. The results showed that total triterpenes and total phenols had anti-lung cancer activity and their combination significantly enhanced the activity. In addition, the combination also significantly increased the TNF-α content compared to total triterpenes or total phenols. The results indicated that the efficacy of *Prunella* against lung cancer was attributable to multiple components acting at an optimal ratio.

## 1. Introduction

The use of herbs for the prevention, treatment and diagnosis of diseases has been practiced for thousands of years in China, Japan, Korea, and other countries [1,2]. The active chemical components of every medicinal herb play a key role in prevention and treatment of diseases [3,4]. However, often not all chemical components in herbs, but only several isolated plant-derived compounds have specific activity for a disease: digoxin, digitoxin, quinine, vinpocetine, vinblastine, sennosides, morphine, codeine, papverine, hiperforine, *etc*. In addition, some components also showed the specific activity, such as polyphenone E [5], ginkgo biloba extract [6], *etc*. For mixtures of compounds, the ratios of these compounds also play a key role in the efficacy of the drug, for example, the antibiotic sulfamethoxazole is often used in combination with trimethoprim in a 5:1 ratio because it then exhibits stronger antibacterial activity [7]. Moreover, determining the optimal ratio of the components is also important for maximizing the efficacy. The extract of ginkgo biloba (Egb) consists of compounds such as flavonoids, terpenoids, organic acids, alkyl and phenol acids and steroids [8]. It was found that each component exists in a certain ratio in the extract and 87% of the composition has been identified, which included 24% of total flavonol glycosides, 6% of terpene lactones, 7.0% of proanthocyanidins, 13.0% carboxylic acids, 2.0% catechins, 20% of non-flavonoid glycosides, 4.0% of polymers, 5.0% of inorganic matter, 3.0% of water and 3.0% other compounds. 

*Prunella* is a herb from the Labiatae family that is indexed in the 2005 edition of the *Chinese Pharmacopoeia* [9]. Its main bioactive compounds were considered to include terpenoids, phenol acids, flavonoids and polysaccharides [10,11]. *Prunella *showed significant activity in the prevention and treatment of lung cancer through antiproliferation, regulation of tumor cell division cycle, promotion of apoptosis, antioxidative effects [12,13], immune regulation [14,15], antimutagenic effects [16,17,18], stimulation of macrophage phagocytic activity, and induction of gene expression and production of macrophage-related cytokines. In our previous study, we found total phenols had strong antioxidative activity and polysaccharides had antitumor activity *in vivo*. In addition, total triterpenes also showed strong cytotoxicity towards A549 cells [11]. Therefore, we speculatesd that total triterpenes, total phenols (including flavonoids) and polysaccharide are the active components of *Prunella *contributing to the prevention of lung cancer. In this study, the chemopreventive activity of Prunella from different regions was compared *in vivo* and *in vitro*. The compounds of *Prunella* from different regions were analyzed by High Performance Liquid Chromatography (HPLC). We found the active components had an optimal ratio for chemoprevention efficacy in lung cancer and proposed the “active components combination model” based on these results. 

## 2. Results and Discussion

### 2.1. Proliferation inhibition activity for A549 and SPC-A-1 cells

**Figure 1:**
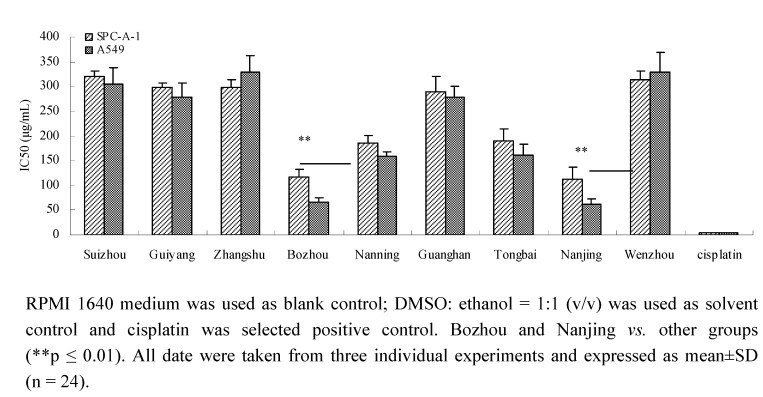
Proliferation inhibition activity of Prunella from different regions on A549 and SPC-A-1 cells.

The 3-(4,5-dimethylthiazol-2-yl)-2,5-diphenyltetrazolium bromide (MTT) method was used for the evaluation of proliferation inhibition activity *in vitro* [19]. As can be seen in Figure 1, there are significant differences between Nanjing and Bozhou *Prunella* and samples from other regions (p ≤ 0.01). These results showed that the inhibitory activities of *Prunella* from Nanjing and Bozhou were the best. Moreover, the results indicated that the inhibitory effect on lung adenocarcinoma cells varied significantly among the *Prunella* samples from different regions. 

### 2.2. Anti-tumor activity in vivo of different Prunella samples in Lewis C57 BL/6 mice

**Figure 2:**
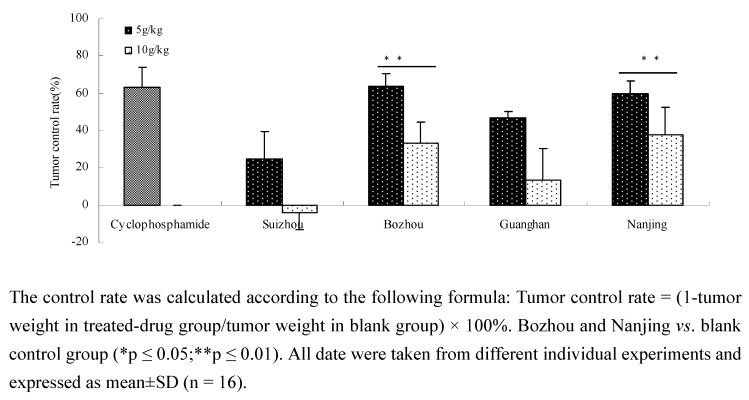
Inhibition activity on lung cancer *in vivo.*

A Lewis C57 BL/6 mice model was used to assess the antitumor activity of different *Prunella* spp. *in vivo* [15]. Based on the *in vitro* screening results, *Prunella* from four regions (Bozhou, Nanjing, Suizhou and Guanghan) were selected for further experiments *in vivo*. As can be seen in Figure 2, the high-dose group of *Prunella* from Nanjing (60.05 ± 6.76%) and Bozhou (63.56 ± 6.79%) was significantly different (p ≤ 0.01) compared with other groups (except the positive group, 62.93 ± 11.05%). This showed that *Prunella* possessed powerful anti-tumor activity and the inhibitory effect of *Prunella* from different regions had significant differences *in vivo*.

### 2.3. Comparison of TNF-α content of Prunella from different regions

**Figure 3:**
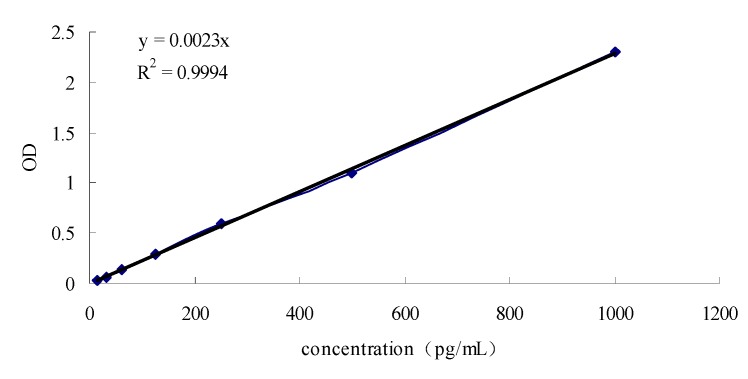
Standard curve of TNF-α.

**Figure 4:**
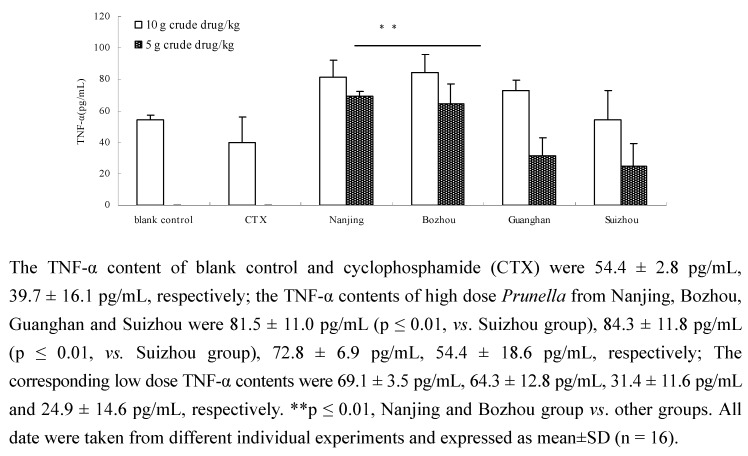
Comparison of TNF-α content in serum of tumor-bearing mice.

TNF-α ELISA kit was used to evaluate the effect of extracts or components on TNF-α content in serum of tumor-bearing mice [20]. TNF-α is a cytokine that has the strongest anti-tumor activity and was found to have significant *in vitro* and *in vivo* cytotoxicity for tumor cells [21]. TNF-α can enhance the efficacy of anticancer drugs [22], inhibit selectively tumor vascular epithelial cells, display anti-angiogenic activity, cause endothelial dysfunction and igh permeability of tumor blood vessels, and induce classical and non-classical caspase-dependent apoptosis [23,24]. In this experiment, OD was highly correlated with the TNF-α content (Figure 3). As shown in Figure 4, there was a significant difference (p ≤ 0.01) between the TNF-α content of the Nanjing and Bozhou groups and the other groups. The results also showed that the Guanghan high dose group enhanced the TNF-α content in serum of tumor-bearing mice. The CTX group showed significantly decreased TNF-α content in the tumor-bearing mice, which may be related to the suppression of immune organs. These results indicated that the TNF-α enhancement ability of *Prunella* from different regions was different.

### 2.4. HPLC analysis

**Figure 5:**
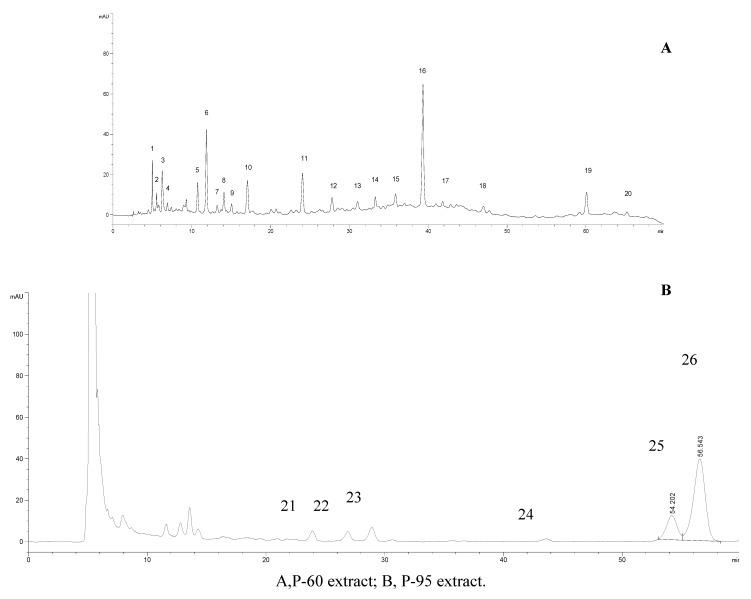
HPLC chromatograms of PV extracts of different *Prunella* samples from different regions.

The chromatograms of extracts of Prunella are shown in Figure 5 and peak areas for the 26 peaks detected in the extract are summarized in Table 1. The data showed that the areas of peaks 9, 11, 16, 17, 18, 22, 23, 25 and 26 from the Bozhou and Nanjing samples were different than those from other regions. In most of these runs, six components could be identified as caffeic acid (peak 11), rosmarinic acid (peak 16), rutin (peak 17), quercetin (peak 18), oleanolic acid (peak 25) and ursolic acid (peak 26) after comparing with their standards; their overall ratios in *Prunella* from Bozhou and Nanjing were found to be similar: 1.0:14.7:3.9:1.0:4.4:1.4 and 1.0:14.8:4.0:0.8:5.6:1.8, respectively. The results suggested that these compounds were associated with efficacy and the ratio played a crucial role in this efficacy.

**Table 1:** Peak area comparison of the 26 compounds in *Prunella* from different regions.

Peak No.	Suizhou	Guiyang	Zhangshu	Bozhou	Nanning	Guanghan	Tongbai	Nanjing	Zhejiang
1	153.8	374.2	268.8	227.8	168.7	305.1	148.0	91.4	247.0
2	83.6	125.5	478.4	303.9	109.4	155.7	102.7	38.4	76.1
3	252.6	808.1	469.8	423.9	276.1	388.9	174.2	197.1	336.1
4	69.1	163.4	150.7	82.2	100.4	126.3	58.7	54.0	43.1
5	151.9	1535.2	123.4	89.5	44.6	85.8	78.3	74.4	162.8
6	488.5	1905.8	1780.2	1648.6	864.9	1374.8	694.2	341.4	961.2
7	59.4	83.2	210.7	149.9	154.7	160.3	78.1	66.9	63.7
8	139.6	408.1	174.4	117.2	217.4	212.7	101.9	182.0	182.1
9	79.6	69.9	126.3	114.2	181.1	142.2	37.4	103.7	87.9
10	249.2	414.4	489.5	386.5	466.2	483.9	227.6	446.2	413
11	63.3	93.9	252.8	148.9	201.6	194.8	248.3	148.3	99.5
12	125.7	20.2	56.8	150.4	66.3	41.7	22.4	21.4	16.0
13	87.6	351.9	170.8	164.2	110.5	28.3	129.4	73.7	124.0
14	102.9	98.3	149.8	143.9	55.5	104.5	97.2	68.3	68.8
15	145.5	566.7	688.3	623.2	292.5	819.9	526.0	434.6	617.4
16	200.3	410.5	2725.8	1558.5	1901.3	1275.9	1399.5	1567.9	1056.4
17	0.00	54.8	496.3	82.7	224.6	114.2	189.4	84.9	52.6
18	57.6	87.8	635.3	106.2	213.6	186.2	91.5	90.4	106.2
19	230.9	1178.5	1221.6	1569.1	723.7	1166.4	461.4	193.2	814.5
20	35.9	45.3	46.6	59.1	42.6	46.8	38.1	47.8	32.5
21	207.9	648.2	284.8	260	155	497.8	440.7	149.8	211.8
22	220.5	352.6	277.1	187.8	175.2	267.8	377.8	175.3	173.5
23	117.4	472.7	315.8	314.7	273	383.5	468.2	302.6	259.8
24	70.1	182.6	262.7	601.2	118.7	223.8	103.2	51.9	146
25	1219.9	1236.1	911.7	901.6	725.2	2093.5	805.4	1110.7	1174.1
26	3417.8	3426.1	2559.3	2452.7	2061.8	5813.9	3465.5	3035.8	3394.6

The all data were taken from three individual experiments and expressed Table 2 as the mean.

**Table 2:** Contents of six compounds in *Prunella* spp. and their ratios (mg/g).

Regions	Species	Caffeic acid	Rosmarinic acid	Rutin	Quercetin	Ursolic acid	Oleanolic acid	Ratio
Suizhou	*Prunella vulgaris *L.	0.04	0.17	_	0.05	0.56	0.18	1.0 **: **4.3 **: **0.0**: **1.3 **: **14.0 **: **4.5
Guiyang	*Prunella vulgaris *L.	0.05	0.35	0.23	0.07	0.56	0.18	1.0 **: **7.0 **: **4.6 **: **1.4 **: **11.2 **: **3.6
Zhangshu	*Prunella vulgaris *L.	0.15	2.31	2.12	0.52	0.42	0.13	1.0 **: **15.4**: **14.1**: **3.5**: **2.8 **: **0.9
Bozhou	*Prunella vulgaris *L.	0.09	1.32	0.35	0.09	0.40	0.13	1.0 **: **14.7 **: **3. 9 **: **1.0 **: **4.4 **:**1.4
Nanning	*Prunella vulgaris *L.	0.12	1.61	0.96	0.18	0.34	0.10	1.0**: **13.4 **: **8.0 **: **1.5 **: **2.8 **: **0.8
Guanghan	*P. Hispida *Bent	0.11	1.18	0.49	0.15	0.95	0.30	1.0 **: **10.7 **: **4.5 **: **1.4 **: **8.6 **: **2.7
Tongbai	*P. Asiatica *Nakai	0.14	1.19	0.81	0.08	0.57	0.12	1.0 **: **8.5 **: **5.8 **: **0.6 **: **4.1 **: **0.9
Nanjing	*P. Asiatica *Nakai	0.09	1.33	0.36	0.07	0.50	0.16	1.0 **: **14.8 **: **4.0 **: **0.8 **: **5.6 **: **1.8
Wenzhou	*P. Asiatica *Nakai	0.06	0.90	0.22	0.09	0.95	0.17	1.0 **: **15.0 **: **3.7 **: **1.5 **: **15.8 **:**2.8

“-” Not detected. The regression equation and correlation coefficients of caffeic acid, rosmarinic acid, rutin, quercetin, ursolic acid and oleanolic acid were *Y *= 28563*X*-4.5, *R *= 1.000;*Y *= 12569*X *- 8.1, *R *= 1.000; *Y* = 4690*X*- 5.5, *R *= 0.9999; *Y* = 24347*X *+ 4.1, *R *= 0.9998; *Y *= 5485.7*X *- 11.2, *R *= 0.9994; *Y* = 6106.4*X* + 17.4, *R *= 0.9996, respectively. The all data were taken from three individual experiments and expressed as the mean.

### 2.5. Cluster analysis

**Figure 6:**
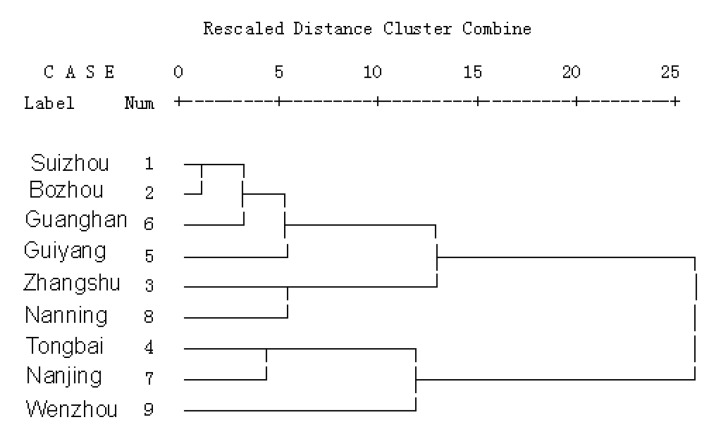
Pedigree chart cluster of *Prunella*.

By cluster analysis (di = 15), *Prunella* from Suizhou, Bozhou, Zhangshu, Guiyang, Nanning and Guanghan were classified as one category, while Tongbai, Nanjing and Wenzhou *Prunella* were classified as a class (Figure 6). To our surprise, the two most active Prunella from Nanjing (*P. asiatica* Nakai) and Bozhou (*P. vulgaris* L.) were not assigned to the same class based on the difference of the 26 peaks. This indicated that not all of the components were associated with anti-tumor activity, but rather some compounds, components or several components. 

### 2.6. Inhibition of components and component combinations on SPC-A-1 cells

**Figure 7:**
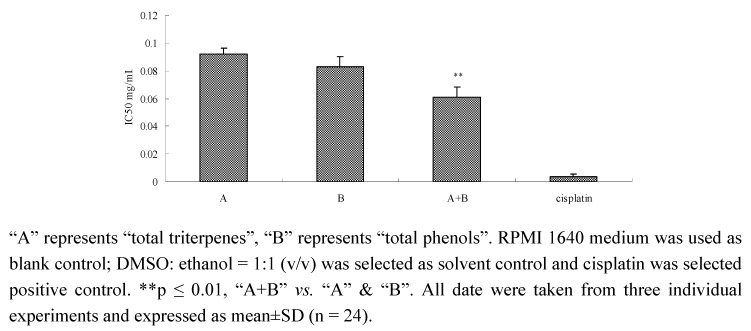
Inhibition ratio of components and its combination on SPC-A-1 cells.

After treatment with components (total triterpenes or total phenols) and mixtures of components for 48 h, the inhibition ratio of SPC-A-1 cells was measured. As shown in Figure 7, there is a significant difference between total triterpenes + total phenols group and other groups (**p ≤ 0.01). The results showed that the inhibition activity of a combination of components on SPC-A-1 cells was significantly increased.

### 2.7. The effects of components and their combination in vivo

**Figure 8:**
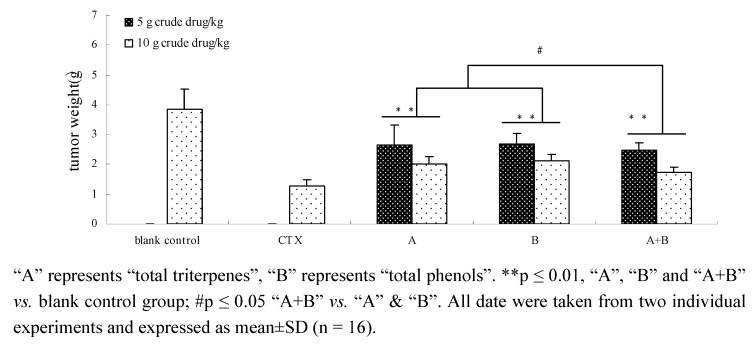
Effect of components and components combination on tumor weight of mices.

**Figure 9:**
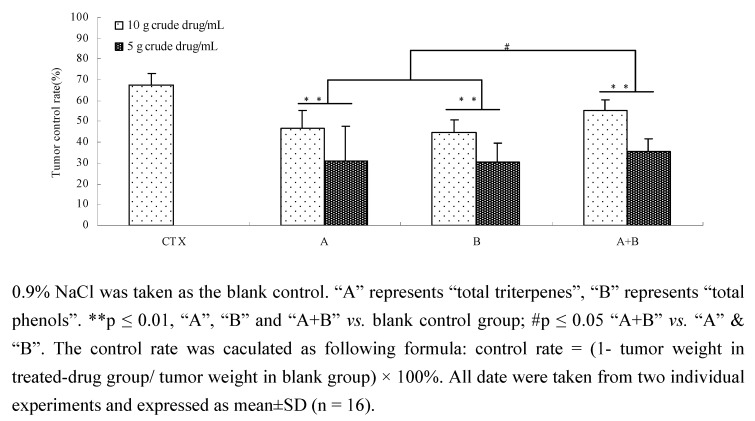
Effect of components and components combination on tumor control ratio of tumor-bearing mice.

As shown in Figure 8 and Figure 9, components (total triterpenes or total phenols) and the combination of components all exhibited strong anti-lung cancer activity in tumor-bearing mice induced by Lewis cells. After treatment with a combination of components, Total triterpenes, total phenols and their combination decreased significantly the tumor weight (p ≤ 0.01) and the tumor control rates (p ≤ 0.01). More importantly, the decreases of tumor weight and tumor control rates in the components combination group were more meaningful compared to the total triterpenes or total phenols groups (p ≤ 0.05). The results indicated that a combination of components could increase anti-tumor activity.

### 2.8. Comparison of TNF-α of components and their combination in vivo

**Figure 10:**
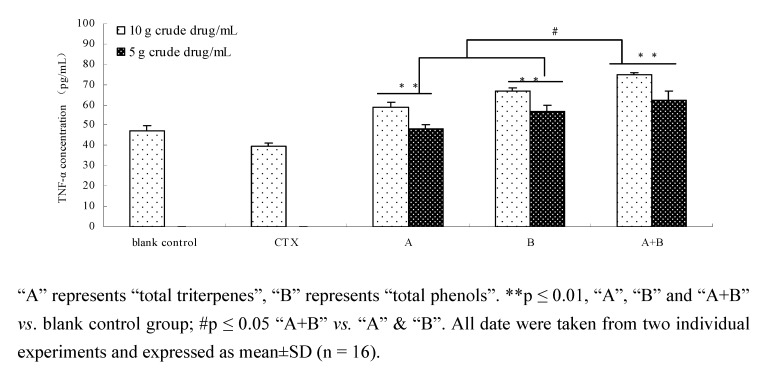
Effect of components and components combination on TNF-α content in serum of tumor-bearing mice.

As seen in Figure 10 repeated, total triterpenes and total phenols components, as well as their combination could significantly increase the TNF-α content in serum of tumor-bearing mice as compared with blank control and CTX control (p ≤ 0.01). The increase of TNF-α content in components combination was higher significantly than for the total triterpenes or total phenols groups (p ≤ 0.05).

Currently, a single compound is often taken as the indicator of quality control for a Chinese herb, which often results in a lack of correlation between efficacy and the herb’s composition. In most cases, although the content of a compound listed in the Chinese materia medica was highest, the efficacy of the compound representing the herb was not the best. Therefore, the differences in efficacy should be determined by the concentration of a number of compounds in nd not by a single compound. Moreover, in the case of *Prunella* the efficacy could be attributed to multiple active compounds at an optimal ratio. 

The efficacy of *Prunella* from different areas varied greatly from the *in vivo* and *in vitro* studies. This referred to genuine regional materia medica, which recognizes the herb with the best efficacy. The difference in efficacy could be caused by the following reasons: the existence of intrinsic differences in genes and other factors such as differences in weather, light, soil and water. Although all *Prunella* samples from different regions contained the same components, their concentrations and ratios were different, thus they have different efficacy. It was found that *Prunella* from Nanjing (*P. Asiatica* Nakai) and Bozhou (*Prunella vulgaris* L.) had the best anti-lung cancer efficacy.

The cluster analysis results indicated that *Prunella* from Nanjing and Bozhou, which have similar anti-tumor activity, were not classified in the same class. This suggested that the efficacy and multiple peaks in fingerprints are very relevant. This might be because only some of the compounds were active and have anti-tumor effects. By comparing the chromatographic peak areas, we found that some peak areas of the *Prunella* samples from Nanjing and Bozhou with the best efficacy and the others were significantly different. We speculate that these peaks correspond to compounds that might be associated with anti-lung cancer activity. This also supports our hypothesis of an “active components combination model”.

## 3. Experimental

### 3.1. General

Dried spikes of different *Prunella* spp. were purchased from different regions in China, which included Suizhou (Hubei), Guiyang (Guizhou), Zhangshu (Jiangxi), Bozhou (Anhui), Nanning (Guangxi), Guanghan (Sichuan), Tongbai (Henan), Nanjing (Jiangsu) and Wenzhou (Zhejiang). Crude herbs were identified by Professor D.K. Wu from Nanjing University of Chinese Medicine. Herbs from Suizhou, Guiyang, Zhangshu, Bozhou and Nanning were *P. vulgaris* L.; the herb from Guanghan was *P. hispida* Benth; herbs from Tongbai, Nanjing and Wenzhou were *P. asiatica* Nakai. The voucher specimens were deposited in Jiangsu Provincial Academy of Chinese Medicine. 3-(4,5-Dimethylthiazol-2-yl)- 2,5-diphenyltetrazolium bromide (MTT) was obtained from Sigma (St. Louis, MO, USA). HPLC grade methanol and acetic acid were (TEDIA, United States) used as mobile phase. Cisplatin and cyclophosphamide were purchased from Sigma. TNF-α assay kit was offered were offered by Wuhan Boster Biological Technology, LTD. (Wuhan, P.R.China). All other materials were from commercial sources.

### 3.2. Preparation of extracts of different regions Prunella

The dried spikes (4 kg) of Prunella were refluxed twice in of 95% ethanol (v/v, 40 L) for 2 h. The filtered residue was refluxed twice and sequentially in 60% ethanol (v/v), 30% ethanol (v/v) and distilled water (40 L). The extracts were concentrated by rotary evaporation at 60 ºC and the concentrates were dried under reduced pressure at 50 ºC to give a 95% ethanol extract (P-95) and a 60% ethanol extract (P-60). P-95 mainly includes triterpenes and P-60 includes total phenols (including flavonoids). The obtained amounts of P-95, P-60, P-30 and P-w were 118.60 g, 179.98 g, 157.06 g and 156.96 g, respectively. 

### 3.3. HPLC analysis of extracts

HPLC analysis was performed on an Agilent 1200 series system (Agilent, Palo Alto, CA, USA), equipped with a diode array detector, a quaternary pump and an automatic sample injector. The system was fitted with an Alltima C_18_ column (4.6 × 250 mm, 5 μm, Alltech, USA). The column temperature was set to 30 ºC. Methanol (A) and 0.5% acetic acid (B) were used for the mobile phase. The elution gradient for P-60 was as follows: 2-15% A and 98-85% B in 5 min, 15-50% A and 85-50% B in 35 min, held at 50% A and 50% B for 10 min, 50-60% A and 50-40% B in 10 min, held at 60% A and 40% B for 5 min and then back to 2% A and 98% B in 5 min, for a total elution time of 70 min at 1 mL/min flow rate. The detection wavelength was at 280 nm and the injection volume was 20 μL. The chromatograms for P-60 are shown in Figure 5A. For P-95 the wavelength was 210 nm and the sample was eluted at 85% A for 30 min (Figure 5B).

### 3.4. Preparation of total triterpenes and total phenolic

P-95 (4g) was added into saturated SP825 macroporous resin (400 g, Mitsubishi Chemical, Japan) and eluted successively with 50% ethanol and 95% ethanol to obtain total triterpenes. P-60 (4g) was added into saturated HP20 macroporous resin (300 g, Mitsubishi Chemical, Japan) to isolate total phenols by eluting with distilled water, 30% ethanol and then 50% ethanol. The eluates were analyzed by HPLC to ensure complete elution. The total triterpenes and total phenolic obtained were 2.1 g and 2.8 g, respectively. 

### 3.5. Cell viability by MTT assay

Human lung adenocarcinoma A549 cells and mice lung adenocarcinoma Lewis cells were provided by NanJing KeyGen Biotech Co. (China). Human lung adenocarcinoma cell line SPC-A-1 was provided by the Institute of Biochemistry and Cell Biology (Shanghai Institute for Biological Sciences, Chinese Academy of Sciences, China). The A549 and SPC-A-1 cells were cultured in RPMI-1640 medium supplemented with FBS (10% v/v), streptomycin (100 U/mL) and penicillin (0.1 mg/mL). The cells (100 μL) were seeded into 96-well plates with 4 × 104 cells/mL each well. After 24 h, the media was removed and cells were treated with RPMI-1640 (90 μL without FBS, then crude extract of *Prunella* (10 μL) at different concentrations (final concentration = 50, 100, 200, 400 μg/mL) were added. The extracts were dissolved in DMSO-ethanol = 1:1 (v/v), Final concentration of organic solvent in the medium was 0.48%. Blank control cells were treated with 0.48% organic solvent and positive control cells were treated with 10 μL cisplatin (final concentration = 20 μg/mL). The cells were incubated in a humidified atmosphere with CO_2_ (5% v/v) at 37 ºC for 48 h. Then, 10 μL of MTT solution (5 mg/mL) was added into each cell to generate formazan and incubated in a humidified atmosphere with CO_2_ (5% v/v) at 37 ºC for 4 h. After removing the RPMI-1640 medium and MTT, 100 μL DMSO was added to dissolve the formazan with 10 min vibration with a shaker (Thermo, USA). The optical density (OD) in the wells was read at 550 nm and 690 nm with a microplate reader.

### 3.6. Anti-tumor activity on lung cancer in vivo

Male C57BL/6 mice, 6–8 weeks of age and weighed approximately 18-20 g, were obtained from Shanghai SLAC Laboratory Animal Co., Ltd (China). The mice were maintained under standard environmental conditions and fed with a standard diet and water *ad libitum*. Primary tumors were induced by subcutaneous (s.c.) injections of 10^6^/mL Lewis cells in 100 μL PBS in the right anterior limb. The mices were given orally daily with a 0.4 mL mixture of P-95, P-60, P-30 and P-w (high dose: 10 g crude drug/kg; low dose: 5 g crude drug /kg) for 14 consecutive days. Positive control mice were injected with 0.2 mL/day cyclophosphamide (20 mg/kg). Blank control mice received saline solution (0.4 mL/day) Orally. Blood (0.5 mL) was taken from the venous plexus in the fossa orbitalis before sacrifice and then centrifuged (3,000 rpm, 15 min) to isolate the serum and stored at -20 ºC.

### 3.7. TNF-α immunoassay

TNF-α in serum supernatants was measured using a commercial mouse enzyme-linked immunosorbent assay (ELISA) kit (Boster, China) with a sensitivity of < 15.6 pg/mL. OD values were measured with a microplate reader at 450 nm.

### 3.8. Statistical analysis

According to the HPLC results, the fingerprints of *Prunella* from different regions were established for cluster analysis. The "Traditional Chinese Medicine chromatogram analysis and data management system" was adopted objectively to obtain information on the various compounds of the characteristic peaks. Cluster analysis was performed with SPSS 16.0 software, using the average distance between classes in order to establish the square Euclidean distance measure. Other data were expressed as means±standard deviation (SD) and analyzed by one-way ANOVA with the SPSS 16.0 software. Differences among means were defined at P < 0.05.

## 4. Conclusions

In this study, hypothesis of enhanced activity by the “active components combination model” was proposed. The anti-lung cancer activities differences of *Prunella* spp. from various regions and HPLC analysis were performed to explore the correlation between efficacy and the active chemical components. These results suggested that total triterpenes and total phenols were the active components and the existence of an optimal ratio of compounds plays an important role in chemoprevention of lung cancer.
